# Characterization of Humoral Immune Responses against Capsid Protein p24 and Transmembrane Glycoprotein gp41 of Human Immunodeficiency Virus Type 1 in China

**DOI:** 10.1371/journal.pone.0165874

**Published:** 2016-11-01

**Authors:** Xiufen Li, Yue Wu, Xuqi Ren, Shuyun Deng, Guifang Hu, Shouyi Yu, Shixing Tang

**Affiliations:** 1 Guangdong Provincial Key Laboratory of Tropical Disease Research, Department of Epidemiology, School of Public Health, Southern Medical University, Guangzhou, Guangdong, China; 2 Department of Sexually Transmitted Diseases, Guangdong Provincial Skin Diseases and STD Control Center, Guangzhou, Guangdong, China; 3 State Key Laboratory of Organ Failure Research, Guangdong Provincial Key Laboratory of Viral Hepatitis Research, Department of Infectious Diseases, Nanfang Hospital, Southern Medical University, Guangzhou, Guangdong, China; China Academy of Chinese Medical Sciences, CHINA

## Abstract

The objective of this study was to extend our previous research and to further characterize the humoral immune responses against HIV-1 p24, gp41 and the specific peptides carrying the immunodominant epitopes (IDEs) that react with human serum samples from HIV-1-infected individuals in China. We found that the majority (90.45%, 180/199) of the samples did not react with any of the three HIV-1 p24 peptides carrying IDEs, but did react with the recombinant full-length p24, suggesting that these samples tested in China were primarily directed against the conformational epitopes of HIV-1 p24. In contrast, 84.54% (164/194) of the samples reacted with at least one HIV-1 linear gp41 peptide, in particular the gp41-p1 peptide (amino acids 560–616). Both recently and long-term HIV-1-infected individuals displayed similar humoral immune responses against the recombinant gp41. However, samples from long-term HIV-1-infected subjects but not from recently infected subjects, showed a very strong reaction against the gp41-p1 peptide. The different response patterns observed for the two groups against the gp41 and the peptide gp41-p1 were statistically significant (*P*<0.01, Chi-square test). These results have direct relevance and importance for design of improved HIV-1 p24 detection assays and the gp41- based immunoassay that can be used to reliably distinguish recent and long-term HIV-1 infection.

## Introduction

During the natural course of human immunodeficiency virus type one (HIV-1) infection, the antibodies specifically recognizing HIV-1 viral proteins are the important components of host humoral immune responses. Among them, the transmembrane portion of HIV-1 envelope protein gp41 is the most conservative antigen that has been widely used for detection of anti-HIV antibodies [1]. The commonly used incidence assays such as the BED-capture-enzyme immunoassay (BED-CEIA) and Limiting antigen avidity enzyme immunoassay (LAg-Avidity EIA) are also based on the proportion of specific anti-HIV gp41 IgG antibodies relative to total IgG [2] or on the affinity of antibodies specifically recognizing the IDEs of HIV-1 gp41 [3]. Neutralizing human monoclonal antibodies (mAbs) like 2F5, 4E10 have also been identified to direct to HIV-1 gp41, making it the primary target for vaccine development [4, 5]. Furthermore, the capsid protein p24 of HIV-1 is the most abundant and highly conserved viral protein and the earliest immunological biomarker detected after HIV-1 infection [6]. The fourth generation HIV-1 immunoassay combines the detection of anti-HIV-1 and anti-HIV-2 antibodies as well as HIV-1 p24 antigen and can further shorten the window period of HIV-1 detection and increase detection sensitivity [7].

Identifying new and recent HIV-1 infections in populations is crucial to prevent HIV transmission, initiate prompt antiretroviral treatment (ART) and monitor the evolution of the HIV-1 epidemic. A more practical approach is to implement incidence assays in a cross-sectional study [8–10]. However, the independent evaluation in specimen panels developed by the consortium for the evaluation and performance of HIV incidence assays (CEPHIA) showed that the current incidence assays did not reach the criteria of large mean duration of recent infection (MDRI) and small false-recent rate (FRR) for distinguishing recent and long-term HIV-1 infection [11]. In fact, overestimations of HIV-1 incidence by BED-CEIA have been repeatedly reported, although the recently modified LAg-Avidity EIA showed a significant improvement in FRR [12–14]. In addition, the current EIA-based incidence assays are time consuming, require special laboratory equipment, and well-trained staff. These limitations prompted the search for novel assays that are quick, inexpensive, easy-to-use, valid, robust, and precise. A new limiting-antigen avidity dot immuno-gold filtration assay for HIV-1 incidence has just been reported to be suitable for point-of-care use [15]. In addition, some new biomarkers for measuring HIV-1 incidence are under investigation and evaluation [16–18].

We previously reported that samples from acutely HIV-1-infected subjects reacted with multiple linear peptides of HIV-1 p24, whereas samples from chronically infected patients reacted with a single peptide or did not react with any linear peptides. We observed a switch of humoral immune response patterns from polyclonal-like reaction during acute HIV-1 infection to monoclonal-like reaction or reaction against conformational epitopes during chronic HIV-1 infection. Based on these findings, we proposed that the specific peptides carrying the major IDEs of HIV-1 p24 may be useful for distinguishing acute and chronic HIV-1 infection [19].

Using recombinant proteins or synthetic overlapping peptides, several B cell epitopes of HIV-1 p24 and gp41 have been determined by testing the immune reaction with the monoclonal antibodies produced by immunized mice or polyclonal antibodies from rabbits or sheep [20]. However, it has been reported that HIV-1 epitopes targeted by the antibodies from human beings or animals were apparently different [21]. Thus, it would be advantageous to use the specific anti-HIV antibodies purified from HIV-1-infected individuals or to determine the immune response patterns in the human specimens taken from infected individuals.

In the present work, we provide further analysis of our previous findings and further characterize the humoral immune response patterns in samples from HIV-1-infected human subjects in China. We have now designed and synthesized three peptides representing major B cell linear IDEs of HIV-1 p24 and gp41, respectively (S1 Table) guided by our previous results [19] and the information from the HIV-1 database (http://www.hiv.lanl.gov/content/immunology/maps/ab.html). In our study, we observed that most of the HIV-1-infected subjects reacted with the recombinant p24 protein, but not with the three linear peptides. There was no significant difference in the humoral immune response patterns against HIV-1 p24 between samples from recently and long-term HIV-1-infected individuals. However, we identified a peptide, gp41-p1, that could react specifically with specimens from long-term infection, but almost not at all from recent infection by HIV-1. The performance of the gp41-p1-based test was further compared with two commercially available LAg-avidity EIA kits. These results are of major importance for development of an improved HIV-1 p24 detection assay and a gp41-based immunoassay to distinguish recent and long-term HIV-1 infection. In turn, this goal is critical for monitoring the spread of the HIV-1 epidemic and initiating appropriate anti-AIDS drug therapy.

## Materials and Methods

### Ethics statement

As mandated by the Declaration of Helsinki, written informed consent was obtained from individuals enrolled in both the cross-sectional and longitudinal studies. The ethical approval was obtained by the Ethics Committee of Guangdong Provincial Skin Diseases and STD Control Center (Guangzhou, China, protocol number 2013-H-01) and Southern Medical University, respectively. This report includes analysis of stored de-linked samples and data from those studies.

### Antigens, peptides, antibodies and HIV-1 testing kits

The recombinant HIV-1 protein p24 and glycoprotein gp41 were purchased from Guangzhou Wondfo Biotech Co., Ltd (Guangzhou, Guangdong, China). Six peptides derived from HIV-1 p24 and gp41 (S1 Table) were synthesized and purified by high performance liquid chromatography (HPLC) at Sangon Biotech Co., Ltd (Shanghai, China). The sources and characteristics of anti-HIV p24 antibodies are listed in the S2 Table. Horseradish peroxidase (HRP) conjugated goat anti-human immunoglobulin G (IgG), mouse anti-human IgG and HRP conjugated goat anti-human λ light chain antibodies were purchased from Abcam (Cambridge, UK). HRP conjugated goat anti-human κ light chain antibody was purchased from Thermo Fisher (Rockford, IL, USA). HRP conjugated goat anti-mouse IgG and goat anti-rabbit IgG were purchased from Bioworld (Minneapolis, MN, USA). HIV-1 Limiting antigen avidity EIA kits were purchased from Maxim Biotech (Rockville, MD, USA) and KingHawk Pharmaceuticals Inc. (Beijing, China) and are called Maxim LAg Avidity EIA Kit and KingHawk LAg Avidity EIA Kit, respectively, in our study.

### Samples

#### Cross-sectional samples

A total of 199 serum or plasma samples were collected from HIV-1-infected individuals including 70 intravenous drug users (IDUs) and 129 men who have sex with men (MSMs) in Guangzhou, China (Table 1). The IDUs and MSMs enrolled in our study did not receive ART. CD4 T-cell counts were not available. Twenty serum samples negative for both anti-HIV-1 and anti-HIV-2 were also collected as negative controls. HIV-1 infection was originally determined by an in-house enzyme-linked immunosorbent assay (ELISA) using gp41 as the coating antigen and was confirmed by RT-PCR and sequencing. The PCR primers and conditions for HIV-1 RNA detection and genotyping have been reported previously [22]. These serum or plasma samples were heat inactivated at 56°C for 30 min before testing. The Maxim and KingHawk HIV-1 LAg-Avidity EIA Kits were used to determine recent or long-term infection by HIV-1 according to the manufacturer’s instructions (S3 Table).

**Table 1:** Characteristics of individuals and samples used in the study.

Groups	Number tested	Classification of HIV-1 Infection		P value
		Recent^b^	Long-term	
Cross-sectional samples				
IDUs	70	1 (1.43%)	69 (98.57%)	<0.05
MSMs	129	37 (28.68%)	92 (71.32%)	
Total	199	38 (19.10%)	161 (80.90%)	
HIV-1 genotypes^a^				
CRF01_AE	56	16 (51.61%)	40 (50.00%)	0.98
CRF07_BC	36	10 (32.26%)	26 (32.50%)	
Subtype B	9	2 (6.45%)	7 (8.75%)	
Others	10	3 (9.68%)	7 (8.75%)	
Total	111	31 (100%)	80 (100%)	
Longitudinal samples				
Average Days postinfection	37	74 ± 27	355 ± 141	<0.05
Average CD4+ T-cell counts (cells/mm^3^)	37	515 ± 171	401 ± 129	<0.05

^a^ HIV-1 genotypes were determined in 111 MSMs.

^b^ Recent and long-term HIV-1 infection was determined by using the Maxim LAg-Avidity Kit.

#### Longitudinal samples

A total of 40 serum samples were collected from 10 HIV-1-infected treatment-naïve MSMs. Each individual provided 4 serum samples at 4 different time points postinfection. The number of days postinfection was estimated according to the records showing the first time HIV-1 seroconversion or detection of HIV-1 RNA occurred. Subjects were followed longitudinally through at most 602 days post-seroconversion. The average follow-up period was 244 ± 175 days postinfection. CD4 cell counts were measured for all the 40 samples. The Maxim and KingHawk HIV-1 LAg-Avidity EIA Kits were used to determine the classification of HIV-1 infection (recent or long-term infection) (S3 Table).

### ELISA for detection of antibodies against recombinant HIV-1 antigens and peptides

The recombinant antigens or synthetic peptides were diluted to a final concentration of 2.5 μg/ml and 5 μg/ml, respectively in 0.01 M phosphate-buffered saline (PBS). Fifty microliters of the coating solution were added to each well of the 96-well plate followed by incubation at 4°C overnight. The plate was washed five times with the PBS containing 0.05% Tween-20 (PBS-T) and then blocked by adding 200 μl of 5% non-fat dry milk (NFDM) diluted with PBS-T at 37°C for 1 h. Anti-HIV-1 antibodies or 1:100 diluted human sera were added and incubated at 37°C for 1 h. After washing with PBS-T, the plate was incubated with HRP conjugated goat anti-human or anti-mouse IgG, or goat anti- rabbit IgG at 37°C for 30 min. Finally, the plate was incubated with 50 μl of 3, 3’, 5, 5’-tetramethylbenzidine (TMB) solution at room temperature for 15 min. The reaction was stopped by addition of 50 μl of 2 M H_2_SO_4_ to each well. Optical density (OD) was read at an absorbance of 450 nm (630 nm as reference). The cut-off (CO) absorbance was the average signal intensity of negative controls plus 2 standard deviations (SD). Samples with S/CO values of ≥ 1.00 were considered positive. To determine which peptides were reactive with the HIV-1-infected samples, the OD value for the antibody against the recombinant p24 or gp41 proteins was taken as 100%, while the OD value for the antibody against the six peptides was expressed relative to that against the recombinant p24 or gp41. A percentage of ≥ 20% was considered positive.

For detection of antibodies against gp41-p1 peptide, the assay conditions were modified as follows: 1) using 1 μg/ml of peptide for coating; 2) after incubation with serum samples, the plate was washed and treated with 4 M urea for 10 min followed by washing 5 times. The rest of the assay procedures were the same as above.

### ELISA for detection of light chain isotypes of human IgG

Microtitre plates were coated with 10 μg/ml of mouse anti-human IgG or 2.5 μg/ml of recombinant HIV-1 p24 or gp41 proteins in 0.01 M PBS (pH 7.4) at 4°C overnight. After washing five times, plates were blocked with 200 μl of 5% NFDM and incubated at room temperature for 1 h. After washing, 1:100 diluted human sera were added and incubated at 37°C for 1 h followed by incubating with HRP conjugated anti-human κ or λ antibodies (1:4000 and 1:500 dilutions, respectively) at 37°C for 30 min. The plate was then washed and developed with TMB solution. The reaction was stopped with 2 M H_2_SO_4_ and read at an absorbance of 450 nm (630 nm as reference). The κ / λ ratio was calculated as OD_κ_ / OD_λ_.

### Statistical analysis

The statistical analysis was conducted using the SPSS 22.0 statistical software package (SPSS Inc., Chicago, IL, USA). Descriptive data were expressed as the mean or as percentage. Qualitative variables were compared using the Chi-square test or Fisher’s exact test. Quantitative variables were compared using the Student's t-test or ANOVA when necessary. A *p* value < 0.05 was considered statistically significant.

## Results

### Characteristics of individuals and samples used in the study

Of the 199 specimens collected from the cross-sectional study, 38 (19.10%) and 161 (80.90%) were classified as recent and long-term infection by HIV-1 Maxim LAg-Avidity EIA Kit (Table 1). Moreover, 97.37% (37/38) of the recent infections were from MSM, indicating that MSM accounted for the majority of recent infections [23]. HIV-1 genotypes were determined in 111 MSMs and equally distributed in recently and long-term HIV-1-infected groups without significant difference (*P* > 0.05, Chi-square test) (Table 1), suggesting that HIV-1 genotypes identified in our study did not affect the classification of recent and long-term HIV-1 infections, although HIV-1 subtype D has been reported to be a confounding factor [12].

Out of the 40 longitudinal samples, 15 and 22 were classified as recent and long-term HIV-1 infections, respectively by the Maxim and KingHawk avidity-based assays, while 3 samples were discordant for the two assays (S3 Table). When the discordant samples were excluded, the average number of days postinfection was 74 ± 27 and 355 ± 141, respectively, for the recently and long-term HIV-1-infected groups. The average CD4 cell counts were 515 ± 171 and 401 ± 129, respectively. The differences observed for days postinfection and CD4 cell counts were statistically significant (Table 1).

### Humoral immune responses against linear peptides and HIV-1 p24 and gp41 recombinant proteins

**Fig 1:**
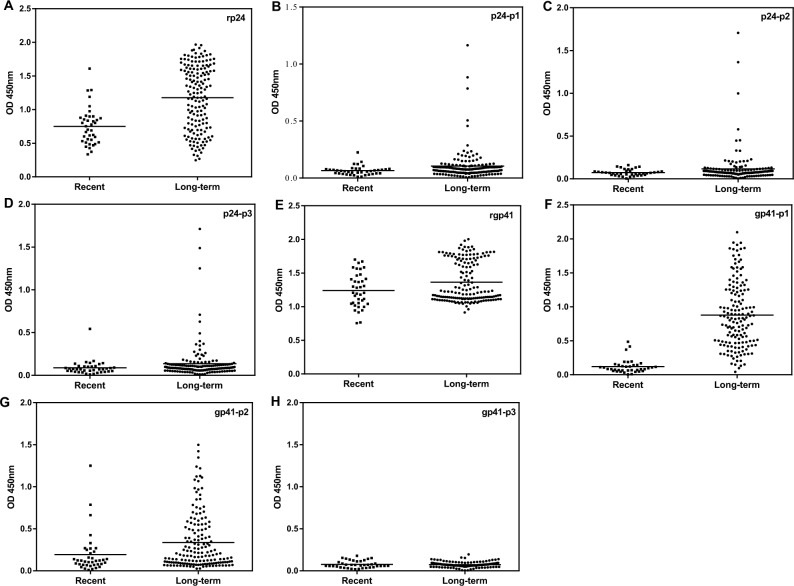
Detection of antibodies against the recombinant p24 or gp41 and peptides in recently and long-term HIV-1-infected individuals. ELISA was used to detect antibodies against recombinant p24 (A), peptide p24-p1 (B), p24-p2 (C), p24-p3 (D), recombinant gp41 (E), peptide gp41-p1 (F), gp41-p2 (G) and gp41-p3 (H), respectively in the samples of HIV-1-infected subjects collected from a cross-sectional study. The antibody titers from each sample are plotted in optical density (OD) on the y-axis. A short line in each plot indicates the mean OD value.

In the cross-sectional study, 199 human serum samples from HIV-1-infected individuals showed strong reactivity against both the recombinant p24 and gp41 proteins. However, the humoral immune response patterns against the linear peptides were quite different (Fig 1, Table 2). We found that the majority (90.45%, 180/199) of the samples did not react with any of the three HIV-1 p24 linear peptides, but did react with the recombinant full-length protein p24 (Table 2), suggesting that these samples were reactive against the conformational epitopes of HIV-1 p24 [19]. Only 19 samples (9.55%, 19/199) were reactive with at least one p24 peptide, including 14 samples that reacted with a single peptide and 5 samples that reacted with two p24 peptides (Table 2). Although abnormal folding of linear peptides may affect serological reaction, this possibility seems unlikely and does not explain the findings in our current study because the HIV-1 p24 peptides and recombinant protein specifically reacted with well-characterized monoclonal and polyclonal antiHIV-1 antibodies (S2 Table). Furthermore, these results are consistent with our previous data in which 9 overlapping peptides covering the entire p24 protein were used and indicate that the current peptide-based assay can specifically distinguish the antibodies against linear or conformational epitopes of HIV-1 p24 [19].

**Table 2:** Humoral immune responses against the peptides or recombinant proteins of HIV-1 p24 and gp41 in the recently and long-term HIV-1-infected individuals.

Parameter	Anti-p24 (%)			Anti-gp41 (%)		
	Recent ^a^	Long-term	Total	Recent	Long-term	Total
**Against one peptide**	2(5.26)	12 (7.45)	14 (7.04)	10(26.32)	76 (48.72)	86 (44.33)
**Against ≥ 2 peptides**	0	5 (3.11)	5 (2.51)	2(5.26)	76 (48.72)	78 (40.21)
**Not reacted with any peptides but reacted with recombinant protein**	36 (94.74)	144 (89.44)	180 (90.45)	26 (68.42)	4 (2.56)	30 (15.46)
**Total**	38 (19.10)	161 (80.90)	199 (100)	38 (19.59)	156 (80.41)	194 (100)

^**a**^ Recent and long-term HIV-1 infection was determined by using the Maxim LAg-Avidity Kit.

In addition to these findings, both recently and long-term HIV-1-infected individuals enrolled in the cross-sectional study displayed similar humoral immune responses against recombinant HIV-1 p24, although the average optical density values for recent infections measured by the antibody detection assay were somewhat lower than the values for long-term infections (Fig 1A). This is likely due to the relatively lower antibody titers and avidity in recent infection compared with chronic infection. About 17 samples from long-term infection were reactive against the p24 linear peptides p1-p3, while only two samples from recently infected subjects were reactive against p24-p3 (Table 2). Regarding the humoral immune response patterns, in particular the reaction against the p24 peptides, no significant differences were observed between samples from recently and long-term HIV-1-infected subjects (Fig 1A–1D) (*P* > 0.05, Fisher’s exact test). These results suggest that HIV-1 p24 and the linear peptides may not be appropriate biomarkers for distinguishing recent and long-term HIV-1 infection.

In contrast, 84.54% (164/194) of the samples reacted with at least one linear gp41 peptide (Table 2). Among them, the gp41-p1 peptide was demonstrated to be dominant, with a positive rate of 78.35% (152/194) in the samples tested (Fig 1F), while 39.18% (76/194) of the samples recognized the gp41-p2 peptide (Fig 1G). Interestingly, no samples were reactive against gp41 peptide p3 (Fig 1H), probably because all the epitopes of gp41-p3 peptide were identified by using mice monoclonal antibodies rather than human antisera (http://www.hiv.lanl.gov/content/immunology/

maps/ab.html). These results indicate that the epitopes recognized by animal and human anti-HIV antibodies may be different [21]. Furthermore, samples from both recent and long-term HIV-1 infection collected from the cross-sectional study displayed similar humoral immune responses against recombinant HIV-1 gp41 (Fig 1E). The majority (95.51%, 149/156) of the samples from long-term HIV-1-infected subjects showed strong reactivity against the peptide gp41-p1 (Fig 1F). However, out of 38 recently HIV-1-infected individuals, only 3 showed weak reactivity against gp41-p1 (Fig 1F). The responses to the gp41-p1 peptide revealed a significant difference between the recent and long-term infections (*P*<0.01, Independent-samples t test), indicating that the gp41-p1 peptide may be useful for distinguishing recent and long-term infection by HIV-1.

**Fig 2:**
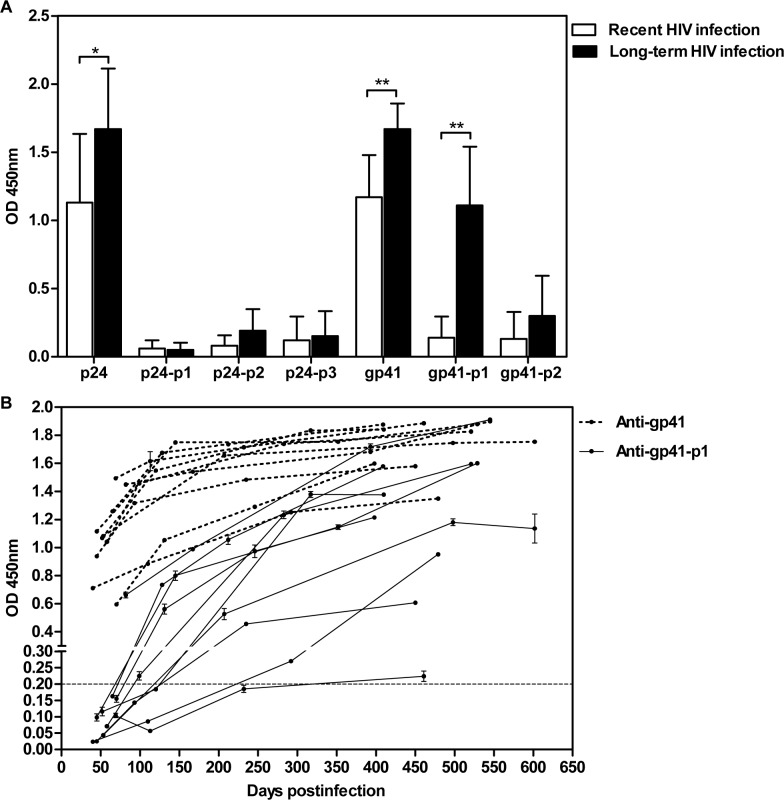
Detection of antibodies against p24 or gp41 recombinant proteins and peptides in the longitudinal samples. The antibody titers are plotted as optical density (OD) on the y-axis. (A) Testing of antibodies against recombinant p24, gp41 and the peptides p24-p1, p24-p2, p24-p3, gp41-p1, gp41-p2 in 37 well-characterized samples from recently (white) or long-term (black) HIV-1-infected subjects. * *P*< 0.05, ** *P*< 0.01. (B) Antibody titers against recombinant gp41 (dotted line) and peptide gp41-p1 (solid line) in the 40 longitudinal sera from 10 HIV-1 infected individuals at different time points. The “recent/long-standing” infection cutoff value is shown by the horizontal dashed line.

The difference between humoral immune response patterns against HIV-1 p24 and gp41, in particular between recent and long-term HIV-1 infection, was further investigated in longitudinal samples (Fig 2A). These samples showed very strong reactivity against HIV-1 recombinant p24, but a weak reaction with three p24 peptides, although the signal intensity for the recent HIV-1 infection was slightly weaker than that for long-term infection (OD values: 1.13 ± 0.51 vs. 1.60 ± 0.48). In addition, a strong immune reaction against gp41 recombinant protein was observed in samples from both recent and long-term HIV-1 infection, whereas there was only a weak immune response directed against the gp41-p2 peptide. However, a dramatic difference in the reactivity against the gp41-p1 peptide was detected in samples from recent and long-term HIV-1-infections (OD values: 0.14 ± 0.15 vs. 1.02 ± 0.48). Fig 2B shows the increasing antibody titers against gp41 and gp41-p1 over various time points postinfection by HIV-1. By ~40 days postinfection, all of the samples tested were strongly reactive against gp41. However, 8 out of 10 HIV-1-infected individuals were seronegative for anti-gp41-p1 until ~120 days postinfection. Only one individual (sample ID3-1) was seropositive against gp41-p1 in his first sample collected at day 82 postinfection, while another individual (ID5-1) was seronegative until day 461 postinfection. These data indicated that by combining the results for reactivity to gp41 and the gp41-p1 peptide, it is possible to distinguish recent and long-term HIV-1 infection. This prompted us to further evaluate the humoral immune response against gp41 and the gp41-p1 peptide as a new method for distinguishing recent and long-term HIV-1 infection.

### Humoral immune responses against gp41 and the gp41-p1 peptide as a new method for distinguishing recent and long-term HIV-1 infection

We used the Maxim and KingHawk HIV-1 avidity-based EIA as the “gold standard” method to test both cross-sectional and longitudinal samples used in our study. The concordance rate of these two commercially available assays was 97.67% (126/129) and 92.5% (37/40), respectively (S3 Table), indicating a comparable testing performance of the two assays. Ultimately, 126 cross-sectional samples and 37 longitudinal samples showed consistent results with the two EIA kits. These samples were then used to further determine the performance of the gp41/gp41-p1-based immunoassay in which anti-gp41 (+)/anti-gp41- p1 (-) and anti-gp41 (+)/anti-gp41-p1 (+) represent recent and long-term HIV-1 infection, respectively (Table 3). Compared with the “gold standard” assays, the concordance rate of our test was 95.24% (120/126) for the cross-sectional samples with a positive predicative vale (PPV) of 89.74% and 97.70% for recent and long-term HIV-1 infection, respectively (Table 3). The performance of our test was further confirmed in longitudinal samples in which the concordance rate of our test was 94.59% (35/37) with a PPV of 100% and 91.67% for recent and long-term HIV-1 infection, respectively (Table 3). The correlation was statistically significant with a κ value (the coefficient of measure of agreement) of 0.887 and 0.885, respectively. Of note, when employing the two avidity-based assays to classify recent and long-term infection of HIV-1, the PPV of our test was slightly better than using the Maxim EIA Kit alone (S4 Table). Due to the limited number of longitudinal samples, it was not possible to calculate the FRR, admittedly a limitation of our current study. Importantly, however, none of the samples from individuals infected > 1 year was misclassified as recently infected in our study (Fig 2B).

**Table 3:** Comparison between gp41 and gp41-p1-based immunoassay and LAg-Avidity EIA for identification of recent and long-term HIV-1 infection.

Samples	Reaction to gp41 and gp41-p1		LAg-Avidity EIA			PPV ^a^ (%)		P value^b^	κ^c^
	gp41	gp41-p1	Recent	Long-term	Total	Recent	Long-term		
**Cross-sectional samples**	+	-	35	4	39	89.74	97.7	0.687	0.887
	+	+	2	85	87				
	**Total**		37	89	126				
**Longitudinal samples**	+	-	13	0	13	100	91.67	0.500	0.885
	+	+	2	22	24				
	**Total**		15	22	37				

^a^ PPV, Positive Predictive Value.

^b^ *P* value was calculated using the McNemar Test.

^c^ κ, the coefficient of measure of agreement.

### Clonally expanded B-cell expression of anti-p24 and gp41 antibodies

**Fig 3:**
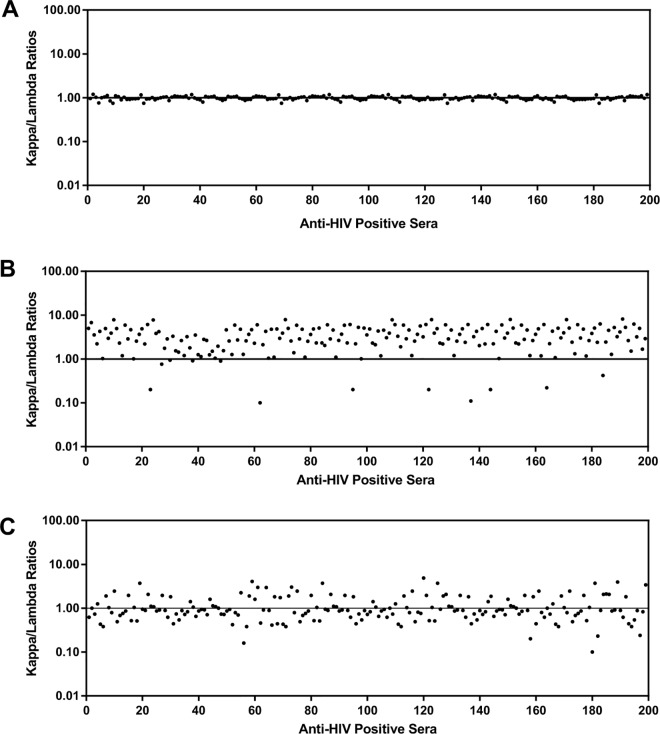
κ / λ ratios in HIV-1-positive sera. Identification numbers of the sera are shown on the x-axis. κ / λ ratios were calculated as described in Materials and Methods. (A) κ / λ ratios for total human IgG. Plates were coated with mouse anti-human IgG and incubated with sera from HIV-1-positive individuals. (B) κ / λ ratios for anti-p24 antibodies. Plates were coated with recombinant HIV-1 p24 and incubated with sera from HIV-1-positive individuals. (C) κ / λ ratios for anti-gp41 antibodies. Plates were coated with recombinant HIV-1 gp41 and incubated with sera from HIV-1-positive individuals.

A unique finding in our study was that 97.48% (194/199) and 59.79% (116/194) of the samples from HIV-1-infected individuals were against the conformational epitopes of HIV-1 p24 or the single linear peptide of HIV-1 gp41, respectively (Table 2). This indicates clonally expanded B-cell expression for both anti-p24 and gp41 antibodies in the HIV-1-infected subjects tested. To further determine the clonal expression patterns of the humoral immune response to HIV-1 p24 and gp41, the contribution of light chain isotypes (κ and λ) was measured in the 199 HIV-1-positive and 20 HIV-1-negative serum samples. To identify the skewed expression of the anti-HIV-1 light chain antibodies, we optimized the assay conditions to maintain the ratio of total human IgG in the serum samples tested to ~1.0. An approximately 1:1 ratio (0.99 ± 0.09) was actually observed in the sample wells coated with anti-human IgG (Fig 3A). However, when the recombinant HIV-1 p24 or gp41 antigen was used to capture the specific antibodies against HIV-1 p24 or gp41, skewed ratios of 3.40 ± 1.90 and 1.15 ± 0.81 were obtained for anti-p24 or anti-gp41, respectively (Fig 3B and 3C). The difference in the light chain ratios between total human IgG and anti-p24 or anti-gp41 was significant in all HIV-1-infected individuals (*P* < 0.001, Paired-samples t test). However, the skewed ratios of light chain use for anti-p24 or anti-gp41 were not significantly different between the recent and long-term infections (*P*>0.05, Independent-samples t test).

Thus, we have characterized the humoral immune response patterns against HIV-1 p24 and gp41 including clonal expansion of B cells secreting anti-HIV antibodies. Moreover, we have identified HIV-1 gp41 as a valuable biomarker for differentiating HIV-1 recent and long-term infection.

## Discussion

HIV-1 p24 is an essential biomarker for early detection and monitoring the progression of HIV-1 infection and has been added in the 4^th^ generation HIV-1 EIA [7]. To improve the assay sensitivity and specificity, it was necessary to identify the major IDEs of HIV-1 p24, in particular in serum samples from HIV-1-infected individuals, since most of the IDEs were previously characterized with monoclonal antibodies generated in mice. We and others have previously identified three major linear IDEs located in the cyclophilin A binding loop and the C-terminal domain as well as the conformational epitopes of HIV-1 p24 [19, 24]. In the current study, we have extended our previous research by investigating the utilization of major HIV-1 p24 IDEs and anti-HIV response patterns in the HIV-1-infected human subjects in China. A unique finding was that 90.45% of the Chinese serum samples were reactive against conformational epitopes rather than the major linear IDEs of HIV-1 p24 (Table 2).

Interestingly, our previous results indicated that only 40% of the samples from the USA. and Cameroon targeted the conformational epitopes of HIV-1 p24 [19]. The obvious difference was not caused by the assay conditions, since the same monoclonal anti-p24 antibodies could specifically recognize the peptides bearing the identical IDEs or conformational epitopes in the two separate studies (S2 Table). One possible explanation for this inconsistency may be the difference in HIV-1 genotypes among the samples tested in the USA, Cameroon, and China. We have reported that no single monoclonal anti-p24 antibody could capture all the main genotypes of HIV-1 [19]. However, the combination of 2 or 3 aniti-p24 antibodies targeted to different epitopes could clearly improve the detection sensitivity and increase the signal strength, suggesting that the subtle amino acid sequence variations and structural changes of HIV-1 strains could result in the difference in antigenicity of HIV-1 p24 and antibody response patterns [19]. The major subtypes of HIV-1 in the USA and Cameroon are subtype B and CRF02_AG, respectively [25], which are quite different from the genotypes identified in China [26, 27]. The majority of the Chinese subjects were infected with HIV-1 subtype B, CRF07_BC, CRF08_BC and CRF01_AE [26, 27]. In our study, MSMs were mainly infected with HIV-1 CRF01_AE (50.45%), CRF07_BC (32.43%), and subtype B (8.11%). However, it remains to be elucidated if the difference of HIV-1 genotypes and variants can explain the difference in humoral immune response patterns that we observed. Moreover, the fact that the overwhelming majority of HIV-1 human samples from China were reactive against p24 conformational epitopes rather than linear epitopes suggests that anti-p24 antibodies targeted to conformational epitopes should be used to improve the detection sensitivity and specificity in the 4^th^ generation HIV-1 EIA in China.

We previously proposed that HIV-1 p24 peptides may be useful for distinguishing acute and chronic infection of HIV-1 since we observed a significant shift in humoral immune response of anti-p24 antibodies, from a polyclonal-like pattern (reacting with multiple linear p24 peptides) during acute infection to a monoclonal-like pattern (reacting with a single peptide) or a pattern against conformational p24 epitopes (not reacting with any linear peptides, but reacting with the recombinant full-length protein) during chronic infection [19]. To further evaluate our findings, the HIV-1-positive sera from China were divided into recent or long-term infection by using the HIV-1 LAg-Avidity EIA Kit recommended by the U.S. Centers for Disease Control and Prevention (CDC). However, in the current study, we did not find any significant difference in the humoral immune response patterns against HIV-1 p24 antigen or peptides between recently and long-term infected individuals (Table 2). Furthermore, more than 90% of the sera tested in our study did not react with any of the three p24 linear peptides carrying the major p24 IDEs, but did react with the p24 protein (Fig 1), comparable to the results reported by Janvier et al. [28]. The results from longitudinal samples were similar (Fig 2A) and surprisingly did not support the use of HIV-1 p24 as a biomarker to distinguish recent and long-term HIV-1 infection.

One explanation for the inconsistent findings may be the difference in the definition of acute and incident HIV-1 infection and the choice of samples. In our previous study, the acutely infected samples were from the American Red Cross and were identified during a longitudinal blood donor screening study [19]. The seroconversion of these acutely infected individuals occurred about two weeks after HIV-1 infection with a median time to seroconversion of 15 days postinfection [19]. This represents an acute phase of HIV-1 infection that typically lasts for 2–4 weeks postinfection [29–31]. In contrast, in our current study, the HIV-1 infection status for these HIV-1 positive samples from a cross-sectional epidemiologic study was determined by the avidity EIA kit. Recent infection was defined as HIV-1 infection occurring in less than 130 days postinfection, according to the manufacturer’s specifications. The current avidity EIA kit cannot determine the exact time of HIV-1 infection.

In general, recent HIV-1 infection is considered to last between 6 and 12 months after infection [32]. Our results suggest that the polyclonal-like humoral immune response patterns against HIV-1 p24 may exist for a very short period following seroconversion, although this hypothesis needs to be verified with a series of follow-up samples. However, we found that several polyclonal rabbit against p24 antibodies (S2 Table) and one pooled HIV-1 infected human sera HIVIG from the NIH AIDS Reagent Program were reactive with a single p24 peptide rather than multiple p24 peptides, indicating clonally expanded B-cell expression of anti-HIV antibodies in both animal immunization and human infection by HIV-1. Dosenovic et al. have observed that the antibody-secreting cells (ASC) against the V3 region of HIV-1 gp120 became dominant at day 3 after the second and third immunization, as measured by the differential B cell ELISPOT assays, indicating a quick affinity maturation of the ASC for the anti-HIV antibodies [33]. Thus, our results suggest that unless the HIV-1 infection is at a very early stage, HIV-1 p24 may not be a reliable biomarker for distinguishing HIV-1 recent and long-term infection.

The humoral immune response patterns against HIV-1 gp41 have also been investigated with the sera from HIV-1 infected individuals and with three linear peptides. Peptides gp41-p1 and gp41-p2 contained the sequences of the cluster I and II regions [34] and could be recognized by about 85% of the samples from HIV-1-infected subjects tested in our study. These results clearly demonstrated that indeed, the gp41-p1 and p2 peptides cover the immunodominant epitopes of gp41 [35]. However, we found that the HIV-1-positive sera did not bind to the third peptide gp41-p3, probably because the epitopes within peptide gp41-p3 were identified by using mouse monoclonal anti-gp41 antibodies and were recognized by animal antisera rather than human sera; however, we could not exclude the possibility of abnormal folding and behavior of the peptide gp41-p3 under the current experimental conditions [35]. The preliminary results indicate that it is critical to rely on the reaction between HIV-1 antigens and human sera to provide authentic identification of the epitopes and humoral immune response patterns.

Unlike HIV-1 p24 antigen, the antibody response induced by HIV-1 gp41 was mainly against the linear epitopes, rather than the conformational epitopes (Table 2). Our finding that the reaction against the gp41-p1 peptide was highly associated with long-term HIV-1 infection is novel. This result is likely due to the relatively weak binding capacity of the short peptide compared with the recombinant full-length gp41 protein as well as the relatively higher affinity of anti-gp41 in chronic HIV-1 infection as opposed to acute infection [2]. Taking advantage of the differential binding capacities of gp41 and the short peptide plus the difference of anti-gp41 affinity and avidity between recent and long-term HIV-1 infection, enabled us to use the combination of gp41 antigen and gp41-p1 peptide to unequivocally distinguish HIV-1 recent and long-term infection. A similar principle has been successfully adapted and approved in the development of the LAg-Avidity EIA and BED-EIA assay [2, 3]; however, our testing method has the advantage of being more straightforward and easy to use. When compared with the available “gold standard” assay Maxim and KingHawk LAg-Avidity EIA, we have observed a concordance rate of 95.24% with the positive predictive value for recent and long-term infection of 89.74% and 97.70%, respectively for the cross-sectional samples. By using longitudinal samples with estimated days postinfection, the performance of our gp41-p1-based assay was further validated and showed a concordance rate of 94.59% compared to the currently used avidity-based assays (Table 3). The preliminary results are very promising and encouraging. In future work, further refinement of the gp41 and peptide gp41-p1 based immunoassay will be needed and independently evaluated with CEPHIA panels to reach the minimum performance characteristics of an MDRI of >120 days and an FRR <2%, as recommended by the WHO/UNAIDS working group on incidence assays [36]. In addition, factors that may be associated with misclassification of HIV-1 recent infection of HIV-1 including viral loads and subtypes as well as CD4+ cell counts must be evaluated [37].

In conclusion, the antibody response patterns against the proteins and peptides of HIV-1 p24 and gp41 were determined by using protein/peptide-based immunoassay of human sera from HIV-1-infected individuals in China. While HIV-1 p24 more likely elicited antibodies against discontinuous conformational epitopes rather than linear epitopes, anti-gp41 antibodies were mainly targeted to the linear epitopes. Importantly, a correlation between the reaction with gp41 single peptide and long-term HIV-1 infection was obtained. These results have direct relevance for the design of improved HIV-1 p24 detection assays and the gp41 based immunoassay for distinguishing recent and long-term HIV-1 infection and are crucial for monitoring the HIV-1 epidemic and determining the appropriate therapeutic strategies for individual AIDS patients.

## Supporting Information

S1 Table Amino acid sequences of the synthetic peptides derived from HIV-1 p24 and gp41. (DOCX)

S2 Table Characterization of anti-HIV-1 p24 antibodies. (DOCX)

S3 Table Comparison between Maxim and KingHawk LAg-Avidity EIA for identification of recent and long-term HIV-1 infection. (DOCX)

S4 Table Comparison between gp41 and gp41-p1-based immunoassay and Maxim LAg-Avidity EIA for identification of recent and long-term HIV-1 infection. (DOCX)

## References

[1] Barin F, McLane MF, Allan JS, Lee TH, Groopman JE, Essex M. Virus envelope protein of HTLV-III represents major target antigen for antibodies in AIDS patients. Science. 1985;228(4703):1094–6. 2986291 10.1126/science.2986291

[2] Parekh BS, Kennedy MS, Dobbs T, Pau CP, Byers R, Green T, et al Quantitative detection of increasing HIV type 1 antibodies after seroconversion: a simple assay for detecting recent HIV infection and estimating incidence. AIDS research and human retroviruses. 2002;18(4):295–307. 10.1089/088922202753472874 11860677

[3] Wei X, Liu X, Dobbs T, Kuehl D, Nkengasong JN, Hu DJ, et al Development of two avidity-based assays to detect recent HIV type 1 seroconversion using a multisubtype gp41 recombinant protein. AIDS research and human retroviruses. 2010;26(1):61–71. 10.1089/aid.2009.0133 20063992

[4] Mehandru S, Wrin T, Galovich J, Stiegler G, Vcelar B, Hurley A, et al Neutralization profiles of newly transmitted human immunodeficiency virus type 1 by monoclonal antibodies 2G12, 2F5, and 4E10. Journal of virology. 2004;78(24):14039–42. 10.1128/JVI.78.24.14039-14042.2004 15564511 PMC533925

[5] Nabel GJ. Immunology. Close to the edge: neutralizing the HIV-1 envelope. Science. 2005;308(5730):1878–9. 10.1126/science.1114854 15976295

[6] Gaines H, von Sydow M, Sonnerborg A, Albert J, Czajkowski J, Pehrson PO, et al Antibody response in primary human immunodeficiency virus infection. Lancet. 1987;1(8544):1249–53. 2884379 10.1016/s0140-6736(87)92696-1

[7] Bentsen C, McLaughlin L, Mitchell E, Ferrera C, Liska S, Myers R, et al Performance evaluation of the Bio-Rad Laboratories GS HIV Combo Ag/Ab EIA, a 4th generation HIV assay for the simultaneous detection of HIV p24 antigen and antibodies to HIV-1 (groups M and O) and HIV-2 in human serum or plasma. Journal of Clinical Virology. 2011;52:S57–S61. 10.1016/j.jcv.2011.09.023 21995929

[8] Serhir B, Hamel D, Doualla-Bell F, Routy JP, Beaulac SN, Legault M, et al Performance of Bio-Rad and Limiting Antigen Avidity Assays in Detecting Recent HIV Infections Using the Quebec Primary HIV-1 Infection Cohort. PLoS One. 2016;11(5):e0156023 10.1371/journal.pone.0156023 27224023 PMC4880343

[9] Mastro TD, Kim AA, Hallett T, Rehle T, Welte A, Laeyendecker O, et al Estimating HIV Incidence in Populations Using Tests for Recent Infection: Issues, Challenges and the Way Forward. Journal of HIV AIDS surveillance & epidemiology. 2010;2(1):1–14. 21743821 PMC3130510

[10] Brookmeyer R, Konikoff J, Laeyendecker O, Eshleman SH. Estimation of HIV incidence using multiple biomarkers. American journal of epidemiology. 2013;177(3):264–72. 10.1093/aje/kws436 23302151 PMC3626051

[11] Kassanjee R, Pilcher CD, Keating SM, Facente SN, McKinney E, Price MA, et al Independent assessment of candidate HIV incidence assays on specimens in the CEPHIA repository. AIDS. 2014;28(16):2439–49. 10.1097/QAD.0000000000000429 25144218 PMC4210690

[12] Longosz AF, Serwadda D, Nalugoda F, Kigozi G, Franco V, Gray RH, et al Impact of HIV subtype on performance of the limiting antigen-avidity enzyme immunoassay, the bio-rad avidity assay, and the BED capture immunoassay in Rakai, Uganda. AIDS research and human retroviruses. 2014;30(4):339–44. 10.1089/AID.2013.0169 24083837 PMC3976571

[13] Hauser A, Santos-Hoevener C, Meixenberger K, Zimmermann R, Somogyi S, Fiedler S, et al Improved testing of recent HIV-1 infections with the BioRad avidity assay compared to the limiting antigen avidity assay and BED Capture enzyme immunoassay: evaluation using reference sample panels from the German Seroconverter Cohort. PLoS One. 2014;9(6):e98038 10.1371/journal.pone.0098038 24892795 PMC4043688

[14] Parekh BS, Hanson DL, Hargrove J, Branson B, Green T, Dobbs T, et al Determination of mean recency period for estimation of HIV type 1 Incidence with the BED-capture EIA in persons infected with diverse subtypes. AIDS research and human retroviruses. 2011;27(3):265–73. 10.1089/aid.2010.0159 20954834

[15] Gao Z, Yan H, Feng X, Wu L, Qiu M, Xing W, et al Development of a New Limiting-Antigen Avidity Dot Immuno-Gold Filtration Assay for HIV-1 Incidence. PLoS One. 2016;11(8):e0161183 10.1371/journal.pone.0161183 27513563 PMC4981313

[16] Wu JW, Patterson-Lomba O, Novitsky V, Pagano M. A Generalized Entropy Measure of Within-Host Viral Diversity for Identifying Recent HIV-1 Infections. Medicine (Baltimore). 2015;94(42):e1865. 26496342 10.1097/MD.0000000000001865 PMC4620842

[17] Moyo S, Wilkinson E, Novitsky V, Vandormael A, Gaseitsiwe S, Essex M, et al Identifying Recent HIV Infections: From Serological Assays to Genomics. Viruses. 2015;7(10):5508–24. 10.3390/v7102887 26512688 PMC4632395

[18] Busch MP, Pilcher CD, Mastro TD, Kaldor J, Vercauteren G, Rodriguez W, et al Beyond detuning: 10 years of progress and new challenges in the development and application of assays for HIV incidence estimation. Aids. 2010;24(18):2763–71. 10.1097/QAD.0b013e32833f1142 20975514

[19] Tang S, Zhao J, Wang A, Viswanath R, Harma H, Little RF, et al Characterization of immune responses to capsid protein p24 of human immunodeficiency virus type 1 and implications for detection. Clin Vaccine Immunol. 2010;17(8):1244–51. 10.1128/CVI.00066-10 20534793 PMC2916249

[20] Liu GJ, Wang JP, Xiao JC, Zhao ZW, Zheng YT. Preparation and characterization of three monoclonal antibodies against HIV-1 p24 capsid protein. Cellular & molecular immunology. 2007;4(3):203–8. 17601374

[21] Liu G, Yang L, Wang J, Zhang G, Chen X, Zheng Y. Immune responses to six synthetic peptides of capsid protein with sera from HIV-1 infected individuals. Cell Mol Immunol. 2005;2(4):289–93. 16274627

[22] Ragupathy V, Zhao J, Wood O, Tang S, Lee S, Nyambi P, et al Identification of new, emerging HIV-1 unique recombinant forms and drug resistant viruses circulating in Cameroon. Virol J. 2011;8:185 10.1186/1743-422X-8-185 21513545 PMC3118203

[23] Friedman DS, O'Byrne P, Roy M. Comparing those diagnosed early versus late in their HIV infection: implications for public health. International journal of STD & AIDS. 2016 8 18 pii: 0956462416664674. 10.1177/0956462416664674 27538724

[24] Janvier B, Archinard P, Mandrand B, Goudeau A, Barin F. Linear B-cell epitopes of the major core protein of human immunodeficiency virus types 1 and 2. Journal of virology. 1990;64(9):4258–63. 1696638 10.1128/jvi.64.9.4258-4263.1990 PMC247891

[25] Hemelaar J, Gouws E, Ghys PD, Osmanov S, WHO-UNAIDS Network for HIV Isolation and Characterisation. Global trends in molecular epidemiology of HIV-1 during 2000–2007. AIDS. 2011;25(5):679–89. 10.1097/QAD.0b013e328342ff93 21297424 PMC3755761

[26] Wang N, Zhong P. Molecular epidemiology of HIV in China: 1985–2015. Zhonghua liu xing bing xue za zhi. 2015;36(6):541–6. 26564620

[27] Chen S, Cai W, He J, Vidal N, Lai C, Guo W, et al Molecular epidemiology of human immunodeficiency virus type 1 in Guangdong province of southern China. PLoS One. 2012;7(11):e48747 10.1371/journal.pone.0048747 23144953 PMC3492446

[28] Janvier B, Lasarte JJ, Sarobe P, Hoebeke J, Baillou-Beaufils A, Borras-Cuesta F, et al B cell epitopes of HIV type 1 p24 capsid protein: a reassessment. AIDS research and human retroviruses. 1996;12(6):519–25. 10.1089/aid.1996.12.519 8679307

[29] McMichael AJ, Borrow P, Tomaras GD, Goonetilleke N, Haynes BF. The immune response during acute HIV-1 infection: clues for vaccine development. Nat Rev Immunol. 2010;10(1):11–23. 10.1038/nri2674 20010788 PMC3119211

[30] Cohen MS, Gay CL, Busch MP, Hecht FM. The detection of acute HIV infection. J Infect Dis. 2010;202 Suppl 2:S270–7. 20846033 10.1086/655651

[31] Powers KA, Miller WC, Pilcher CD, Mapanje C, Martinson FE, Fiscus SA, et al Improved detection of acute HIV-1 infection in sub-Saharan Africa: development of a risk score algorithm. Aids. 2007;21(16):2237–42. 10.1097/QAD.0b013e3282f08b4d 18090052 PMC2673577

[32] Rosenberg NE, Pilcher CD, Busch MP, Cohen MS. How can we better identify early HIV infections? Curr Opin Hiv Aids. 2015;10(1):61–8. 10.1097/COH.0000000000000121 25389806 PMC4490585

[33] Dosenovic P, Chakrabarti B, Soldemo M, Douagi I, Forsell MN, Li Y, et al Selective expansion of HIV-1 envelope glycoprotein-specific B cell subsets recognizing distinct structural elements following immunization. J Immunol. 2009;183(5):3373–82. 10.4049/jimmunol.0900407 19696434

[34] Xu JY, Gorny MK, Palker T, Karwowska S, Zolla-Pazner S. Epitope mapping of two immunodominant domains of gp41, the transmembrane protein of human immunodeficiency virus type 1, using ten human monoclonal antibodies. Journal of virology. 1991;65(9):4832–8. 1714520 10.1128/jvi.65.9.4832-4838.1991 PMC248941

[35] Yusim K, Korber BT, Brander C, Barouch D, de Boer R, Haynes BF, et al. HIV Molecular Immunology 2015. Los Alamos (NM): Los Alamos National Laboratory (LANL); 2016 Apr. Report No.: LA-UR-16—22283. Contract No.: AC52-06NA25396.

[36] Incidence Assay Critical Path Working Group. More and better information to tackle HIV epidemics: towards improved HIV incidence assays. PLoS Med. 2011;8(6):e1001045 10.1371/journal.pmed.1001045 21731474 PMC3114871

[37] Kirkpatrick AR, Patel EU, Celum CL, Moore RD, Blankson JN, Mehta SH, et al Development and Evaluation of a Modified Fourth-Generation Human Immunodeficiency Virus Enzyme Immunoassay for Cross-Sectional Incidence Estimation in Clade B Populations. AIDS research and human retroviruses. 2016;32(8):756–62. 10.1089/AID.2015.0198 26988426 PMC4971410

