# Supplementary material for: Characterization of Humoral Immune Responses against Capsid Protein p24 and Transmembrane Glycoprotein gp41 of Human Immunodeficiency Virus Type 1 in China
Source: PLoS One. 2016 Nov 1;11(11):e0165874. doi: 10.1371/journal.pone.0165874 (PMC5089721; doi:10.1371/journal.pone.0165874)
Supplement: S1 Table — (DOCX) [file pone.0165874.s001.docx]

**S1 Table. Amino acid sequences of the synthetic peptides derived from HIV-1 p24 and gp41**

| **Protein** | **Peptide** | **Sequence** | **Position^a^** |
| --- | --- | --- | --- |
| **p24** | p24-p1 | EGATPQDLNTMLNTVGGHQAAMQMLKDTINEEAAEWDRLHPVHAGP | 177-222 |
|  | p24-p2 | VSILDIRQGPKEPFRDYVDRFFKTLRAEQATQEVKNWMTD | 280-319 |
|  | p24-p3 | TLLVQNANPDCKTILKALGPGATLEEMMTACQGVGGPGHK | 320-359 |
| **gp41** | gp41-p1 | EAQQHLLQLTVWGIKQLQARVLAVERYLKDQKFLGLWGCSGKIICTTAVP WNSSWSN | 560-616 |
|  | gp41-p2 | TSQIYEILTESQNQQDRNEKDLLELDKWASLWNWFDITNWLWYIKIFI | 639-686 |
|  | gp41-p3 | GYSPLSFQTPTHHQREPDRPEEIGEGGGEQGRDRSVRLVS | 718-750 |

^a^ Positions of p24-p1, p24-p2, p24-p3 were derived from intact gag. Positions of gp41-p1, gp41-p2, gp41-p3 were derived from intact gp160. Location of the peptides were taken from the HXB2 isolate of HIV-1 (GenBank accession no. K03455).
