# Supplementary material for: Characterization of Humoral Immune Responses against Capsid Protein p24 and Transmembrane Glycoprotein gp41 of Human Immunodeficiency Virus Type 1 in China
Source: PLoS One. 2016 Nov 1;11(11):e0165874. doi: 10.1371/journal.pone.0165874 (PMC5089721; doi:10.1371/journal.pone.0165874)
Supplement: S2 Table — (DOCX) [file pone.0165874.s002.docx]

**S2 Table. Characterization of anti-HIV-1 p24 antibodies**

| **Antibody ID** | **Vendor** | **Immunogen** | **Clonality** | **Reacting peptides in this study (aa)^a^** | **Reacting peptides in previous study (aa)** |
| --- | --- | --- | --- | --- | --- |
| **3537** | NIH AIDS Research and Reference Reagent Program | Unknown | monoclonal | p2(280-319) | p8(280-329) |
| **C65690M** | Meridian Life Science, Inc. | Unknown | monoclonal | None^b^ | None |
| **1103** | Immuno Diagnostics, Inc. | HIV-1 IIIB p24 | monoclonal | None | None |
| **IT-001-009M1** | Immuno Diagnostics, Inc. | Unknown | monoclonal | None | ND^c^ |
| **IT-001-016** | Immuno Diagnostics, Inc. | HIV-1 clade A p24 | polyclonal | p3(320-359) | ND |
| **IT-001-017** | Immuno Diagnostics, Inc. | HIV-1 clade B p24 | polyclonal | p3(320-359) | ND |
| **IT-001-018** | Immuno Diagnostics, Inc. | HIV-1 clade C p24 | polyclonal | p3(320-359) | ND |

^a^ Location of the peptides were taken from the HXB2 isolate of HIV-1 (GenBank accession no. K03455).

^b^ None, did not react to any of the peptides, but reacted to recombinant p24.

^c^ ND, not done.
