# Supplementary material for: Characterization of Humoral Immune Responses against Capsid Protein p24 and Transmembrane Glycoprotein gp41 of Human Immunodeficiency Virus Type 1 in China
Source: PLoS One. 2016 Nov 1;11(11):e0165874. doi: 10.1371/journal.pone.0165874 (PMC5089721; doi:10.1371/journal.pone.0165874)
Supplement: S3 Table — (DOCX) [file pone.0165874.s003.docx]

**S3 Table. Comparison between Maxim and KingHawk LAg-Avidity EIA for identification of recent and long-term HIV-1 infection**

| **Groups** | **KingHawk** | **Maxim** | | | **P value ^a^** | **κ^b^** |
| --- | --- | --- | --- | --- | --- | --- |
|  |  | **Recent** | **Long-term** | **Total** |  |  |
| **Cross-sectional samples** | **Recent** | 37 | 2 | 39 | > 0.05 | 0.944 |
|  | **Long-term** | 1 | 89 | 90 |  |  |
|  | **Total** | 38 | 91 | 129 |  |  |
| **Longitudinal samples** | **Recent** | 15 | 0 | 15 | > 0.05 | 0.846 |
|  | **Long-term** | 3 | 22 | 25 |  |  |
|  | **Total** | 18 | 22 | 40 |  |  |

^a^ P value was calculated using the McNemar Test.

^b^ κ, the coefficient of measure of agreement.
