# Supplementary material for: Characterization of Humoral Immune Responses against Capsid Protein p24 and Transmembrane Glycoprotein gp41 of Human Immunodeficiency Virus Type 1 in China
Source: PLoS One. 2016 Nov 1;11(11):e0165874. doi: 10.1371/journal.pone.0165874 (PMC5089721; doi:10.1371/journal.pone.0165874)
Supplement: S4 Table — (DOCX) [file pone.0165874.s004.docx]

**S4 Table. Comparison between gp41 and gp41-p1-based immunoassay and Maxim LAg-Avidity EIA for identification of recent and long-term HIV-1 infection**

| **Reaction to gp41 and gp41-p1** | | **Maxim LAg-Avidity EIA** | | | **PPV ^a^ (%)** | | **P value^b^** | **κ^c^** |
| --- | --- | --- | --- | --- | --- | --- | --- | --- |
| **gp41** | **gp41-p1** | **Recent** | **Long-term** | **Total** | **Recent** | **Long-term** |  |  |
| + | - | 35 | 6 | 41 | 85.37 | 98.04 | >0.05 | 0.857 |
| + | + | 3 | 150 | 153 |  |  |  |  |
| **Total** | | 38 | 156 | 194 |  |  |  |  |

^a^ PPV, Positive Predictive Value.

^b^ P value was calculated using the McNemar Test.

^c^ κ, the coefficient of measure of agreement.
